# Gel Stability of Calcium Bentonite Suspension in Brine and Its Application in Water-Based Drilling Fluids

**DOI:** 10.3390/gels8100643

**Published:** 2022-10-10

**Authors:** Zhenhua Zhao, Sinan Chen, Fengshan Zhou, Zhongjin Wei

**Affiliations:** Beijing Key Laboratory of Materials Utilization of Nonmetallic Minerals and Solid Wastes (National Laboratory of Mineral Materials), School of Materials Science and Technology, China University of Geosciences (Beijing), Beijing 100083, China

**Keywords:** calcium bentonite, composite salt-resistant polymer thickening agent, cross linked natural vegetable gum (MVG), salt-resistant polymer mixture (SNV), rheology and stability of an aqueous suspension, drilling fluids

## Abstract

With the development of the oil industry and the increasingly complex drilling environment, the performance of drilling fluids has to be constantly improved. In order to solve the problem of bentonite dispersion and hydration in a saline medium, a drilling fluid additive with good performance and acceptable cost was sought. The effects of several water-soluble polymers, such as cellulose polymers, synthetic polymers and natural polymers, on the rheology and gel suspension stability of calcium-based bentonite were compared in this study. Among the examined polymers, the xanthan gum biopolymer (XC) was the least negatively affected in the saline medium used. However, its high price limits its industrial application in oil and gas drilling fluids. In this study, a salt-tolerant polymer, modified vegetable gum (MVG), was prepared by a cross-linking modification of a natural plant gum, which is abundant and cheap. Then, a salt-tolerant polymer mixture called SNV was prepared, composed of the salt-resistant natural polymer MVG and the biopolymer XC. The salt tolerance and slurry ability of SNV and common water-soluble polymers were evaluated and compared. We then selected the most suitable Herschel–Bulkley model to fit the rheological curve of the SNV–bentonite aqueous suspension system. SNV improved the rheological properties of the calcium-based bentonite slurry and the dispersion stability of bentonite. In an SNV concentration of 0.35%, the apparent viscosity (AV) of the base slurry increased from 2 mPa·s to 32 mPa·s, and the low shear reading value at 3 rpm increased from 0 dia to 5 dia. This could greatly improve the viscosity and cutting carrying capacity of the bentonite drilling fluid. The bentonite drilling fluid prepared with SNV could be directly slurried with brine and even seawater; this means that when drilling in ocean, coastal saline water and high-salinity-surface saline water areas, the slurry preparation cost and preparation time can be conveniently reduced.

## 1. Introduction

Bentonite is a non-metallic mineral resource with high economic value and a wide range of applications. It is a layered silicate mineral mainly composed of montmorillonite, which mainly determines its properties. It has surface electrical properties, strong adsorption capacity and ion exchange capacity. In addition, it can absorb water, has convenient rheology, plasticity and bonding properties and is non-toxic, non-hazardous and insoluble in water. Bentonite has a wide range of uses; currently its three main applications are in drilling fluid slurries, casting and iron ore pellets. In addition, it can be used as an adsorbent to treat wastewater, and in the fields of agriculture and animal husbandry as a soil conditioner, fertilizer additive, etc. [1,2]. Therefore, bentonite is also praised as a universal clay. Bentonite is the most commonly used water-based drilling fluid slurry material in the drilling field; it can cool and flush drill bits, promote borehole stability, carry and suspend drilling cuttings, adjust drilling fluid density, and improve fluid viscosity.

China is the country with the most abundant bentonite resources in the world, with proven reserves of more than 5 billion tons and prospective resource reserves of more than 8 billion tons, accounting for about 60% of the world’s total bentonite reserves. It ranks first in the world for bentonite production, which, however, is mainly low-grade calcium bentonite. Compared with sodium-based bentonite, calcium-based bentonite has poor water absorption properties, swelling capacity and cation exchange capacity; in addition, sodium-based bentonite has better dispersibility, suspension thixotropy, viscosity and lubricity than calcium-based bentonite. Therefore, calcium-based bentonite generally needs to be purified, sodiumized [3] and organically modified to meet the technical requirements of petroleum drilling fluids [4].

With the development of the petroleum industry and the increasingly complex drilling environment, the performance of drilling fluids has to be improved. China is gradually more and more oriented towards offshore drilling, subsea resource development and utilization and deep-sea oil exploration. Unlike onshore oil fields, offshore oil fields use drilling fluids that are mainly configured by seawater and therefore will be contaminated by electrolyte cations. These cations will enter the clay particles through ion exchange and electrostatic forces, compress the double electron layer of the clay particles, lower the zeta potential and thin the hydration film on the surface of the clay particles, thus causing the particles to agglomerate and settle, affecting the rheology, filtration performance, wall building and pH value of the drilling fluid [5,6]. As a result, accidents such as leakage, borehole enlargement, formation collapse, stuck drilling and wellbore instability occur [7].

The Trenchless Technology (TT) emerged in the late 1970s in western developed countries and gradually evolved. It is a new construction technology that uses the technical means of geotechnical drilling to lay, replace or repair various underground pipelines with minimal or no excavation. In recent years, with the rapid development of China economy, the trenchless technology has developed rapidly in China and is now widely used in crossing highways, railways, buildings, rivers, lakes, urban areas, historic sites, protected areas, crop areas or underground pipelines such as those for sewage, tap water, heating, gas, electricity, telecommunications, oil, and natural gas, constructed in vegetation protection areas. Horizontal Directional Drilling (HDD) is one of many trenchless construction technologies [8]. It has become the most widely used and fastest-growing trenchless construction method due to its high construction accuracy and wide application range. In horizontal crossing projects in China, the length of the pipelines laid with the HDD technology exceeded 10,000 km, and the number of HDD-specific drilling rigs exceeded 20,000 by the end of 2019. When using HDD or during coastal geological engineering drilling construction, the drilling fluid must often be prepared with salt water or even sea water.

When the bentonite drilling fluid is used as a seawater drilling fluid system, in order to reduce the influence of high-valent metal ions in seawater on the rheology of the drilling fluid, certain measures must be taken to protect the stability of the drilling fluid hydrogel system. According to previous research results, sepiolite and attapulgite clay minerals can be fully dispersed in a brine medium, which can ensure the stability of the drilling fluid suspension system [9,10].

Sepiolite is a magnesium-rich fibrous mineral with the general chemical formula 4MgO·6SiO_2_H_2_O. The sepiolite drilling fluid has high electrolyte tolerance, good thermal stability, high static shear force and good shear stability. Its suspending ability is hardly affected by the electrolyte. Therefore, sepiolite is a high-quality raw material for drilling fluid preparation. Experiments have shown that an inferior bentonite mud forms a strong colloid with poor rheological properties at 150 °C (the so-called colloid high-temperature curing), which may prevent carrying out the drilling. In contrast, the water-based drilling fluid configured with sepiolite remains stable in suspension even at 200 °C and exhibits salt and pollution resistance [11]. A certain amount of sepiolite can be added to freshwater and brine bentonite drilling fluids as a tackifier and shear-enhancing agent to improve the plastic viscosity, shear force and rock-carrying ability of the drilling fluids [12]. The addition of sepiolite to a drilling fluid can improve the colloidal stability of water-based drilling fluid in brine, but the drilling fluid filter cake structure formed by its fibrous structure has an extremely low density, which causes a filtration loss of more than 100 mL/30 min, can seriously affect borehole safety and damage oil and gas reservoirs. In order to reduce the filtration loss of a drilling fluid to below 15 mL/30 min, large amounts of expensive polymeric agents for filtration loss reduction are required. Therefore, economically speaking, it is not worthwhile to replace bentonite with sepiolite in a seawater drilling fluid.

To solve the problem of reduced bentonite hydration in brine media, two technical countermeasures are often adopted in drilling construction sites: one consists in using freshwater from a nearby area to prepare a freshwater bentonite drilling fluid, gradually adding it to bentonite so to obtain a seawater drilling fluid; the other process consists in applying water softening measures for the precipitation and removal of high-valent metal ions from seawater (usually by adding sodium carbonate, so that calcium and magnesium ions in seawater form insoluble calcium and magnesium carbonates and precipitate). Then, the softened seawater is used to prepare a freshwater drilling fluid. Both processes have the obvious drawback of wasting time and a lot of money. However, the first method may require hauling freshwater from a long distance, with a high hauling cost, and may not be able to timely supply the drilling fluid needed. Although pre-desalinated seawater is removed by adjusting the pH of sodium carbonate to precipitate calcium and magnesium ions, monovalent sodium and potassium ions are still retained in the slurry water, which still has a great adverse effect on the hydration of bentonite [13].

In addition to these two obviously flawed solutions, a third method involves the use of salt-resistant polymer additives to obtain a sodium–calcium-based bentonite (the so-called salt-resistant bentonite, STB) which can be directly used to prepare a bentonite drilling fluid with salt water or even sea water [14]. This method is simple and convenient, greatly improves the efficiency of drilling fluid preparation and the performance of the brine drilling fluid and allows reducing the cost of the brine drilling fluid or seawater drilling fluid [15].

The treatment agents used to prepare drilling fluids directly with seawater can be divided into two categories: organic treatment agents and inorganic treatment agents. Inorganic treatment agents are usually sodium carbonate, sodium hydroxide, potassium hydroxide, etc., used to adjust the pH of the drilling fluid; commonly used organic treatment agents are polyacrylamide (PAM), hydrolyzed sodium polyacrylonitrile (NaPAN), carboxymethyl cellulose sodium (CMC) [16,17], hydroxyethyl cellulose (HEC), polyanionic cellulose (PAC), xanthan gum biopolymer (XC) [18,19,20], sulfomethylated phenolic resin (SMP) and other salt-resistant water-soluble polymers [21,22,23,24,25,26,27].

In order to further enhance the application value of calcium-based bentonite, this paper aimed to identify a suitable anti-salt treatment agent to improve the rheological properties and stability of bentonite in seawater. A treatment agent with excellent performance and low cost may solve engineering problems such as the fact that the bentonite drilling fluid in seawater cannot be directly dispensed, slurry viscosity reduction, and sink destabilization.

## 2. Results and Discussion

In this study, the effects of three types of polymers (cellulose, synthetic and natural polymers) on the gel stability and rheology of calcium bentonite suspensions in brine were investigated by comparative experiments. The linear polymer spread out in the slurry, dispersing the clay particles. The weak gel formed by the polymer and the mud in brine could improve the viscosity and stability of the bentonite suspension and prevent the agglomeration and settlement of bentonite particles.

The salt-resistant polymer was obtained by compounding polymers with excellent performance and high economic value, which can be used as tackifiers and dispersants for seawater-based drilling fluids.

### 2.1. Effect of Cellulose on the Rheology and Suspension Stability of Bentonite

Carboxymethyl cellulose (CMC) and hydroxypropyl methyl cellulose (HPMC) were tested [28].

The two cellulose polymers were mixed with bentonite and Na_2_CO_3_ and added to sea salt water, and the mixture viscosity was recorded with a six-speed viscometer, as shown in Table 1. The slurry with 0.5% cellulose was compared with the base slurry, and it was found that the slurry rheology was significantly improved by cellulose. Its AV was greatly improved. The viscosity-increasing effect of HPMC was obviously greater than that of CMC. After 16 h, we observed a solid–liquid stratification. The clay particles still settled but compared with the base slurry, the stability of the mixture was significantly improved by the addition of the polymer.

### 2.2. Effects of Synthetic Polymers on the Rheology and Suspension Stability of Bentonite

We selected the synthetic polymers zwitterionic polymer (P-1), polycarboxylate water reducer (PAS) and AA\AM\AMPS terpolymer (named A7) [29].

The three synthetic polymers were mixed with bentonite and Na_2_CO_3_ and added to sea salt water, and viscosity was recorded with a six-speed viscometer, as shown in Table 2. After adding 0.5% of each synthetic polymer, we found that, compared with the base slurry, the three polymers had different effects on the slurry rheology. The viscosity-increasing effect of P-1 was obvious, cross-linking occurred in the slurry, and the slurry did not show a solid–liquid stratification. The viscosity-increasing effect of PAS was not obvious, the slurry became a two-phase solid–liquid system, but clay particles remained suspended in the water layer. This indicated that PAS could disperse the clay particles, but because of its low molecular weight, most of the clay particles aggregated and sank. A7 could also increase the viscosity of the slurry. After 16 h, the system did not show delamination.

### 2.3. Effects of Natural Polymers on the Rheology and Suspension Stability of Bentonite

The natural macromolecular polymers selected were xanthan gum (XC) [30], natural modified polymer (P-H) and vegetable gum (MVG). MVG is a polymeric material containing a polysaccharide such as galactomannose as the main component, which is extracted from the roots, stems and leaves of woody plants or herbs [31].

The three natural macromolecular polymers were mixed with bentonite and Na_2_CO_3_ and then added to sea salt water, and viscosity was recorded using a six-speed viscometer. After adding each natural high-molecular-weight polymer at a concentration of 0.5%, we found that, compared with the base slurry, the rheology and stability of the slurry significantly improved. After adding the three polymers, the AV of the slurry increased to more than 20 mPa·s and remained stable for more than 16 h.

### 2.4. Comparison of the Properties of the Three Polymers

**Figure 1:**
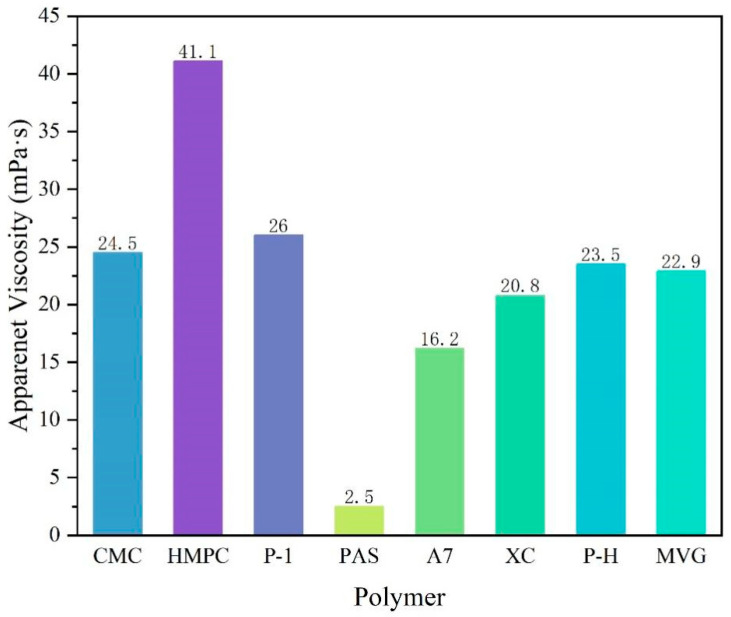
Apparent viscosity of the sea salt slurries containing 0.5% of the examined polymers.

**Figure 2:**
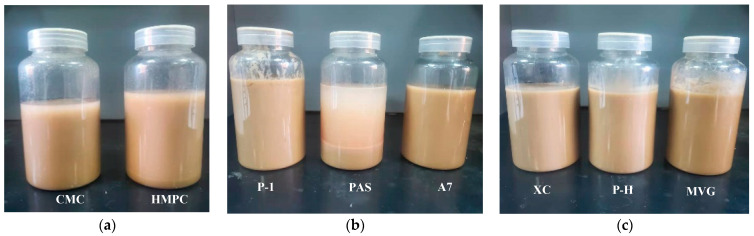
Delamination of polymer and calcium bentonite into sea water suspensions for 16 h. (**a**) Cellulose polymer, (**b**) synthetic polymer, (**c**) natural macromolecule polymer.

A comparison of the apparent viscosities of the bentonite-formulated slurries after the addition of the three polymers is shown in Figure 1. The stratification of polymer and calcium-based bentonite configured into a sea salt water suspension after 16 h is shown in Figure 2. The cellulose polymers produced the best viscosity enhancement for the slurry, but the resulting slurries showed the phenomenon of water and soil stratification. In regard to the synthetic polymers, PAS is a polycarboxylate water reducer for cement, mainly composed of acrylic acid and macromonomers. It can stabilize clay particles in a slurry. However, PAS has a generally short main chain; therefore, the addition of 0.5% PAS will cause water and soil stratification. A7 is a linear polymer obtained by polymerizing three monomers, i.e., AA, AM and AMPS, by aqueous solution polymerization. Its molecular chain is long and carries a sulfonic acid group with strong resistance to salt. Therefore, it can improve a slurry viscosity and stabilize it. P-1 is a cross-linked polymer that is more viscous than linear polymers in an aqueous solution. The natural high-molecular-weight polymer had a very obvious viscosifying effect on the slurry, and it did not cause the stratification of soil and water, even after a long time.

### 2.5. Viscosity Test of Solutions of the Natural Polymers at Different pH

**Figure 3:**
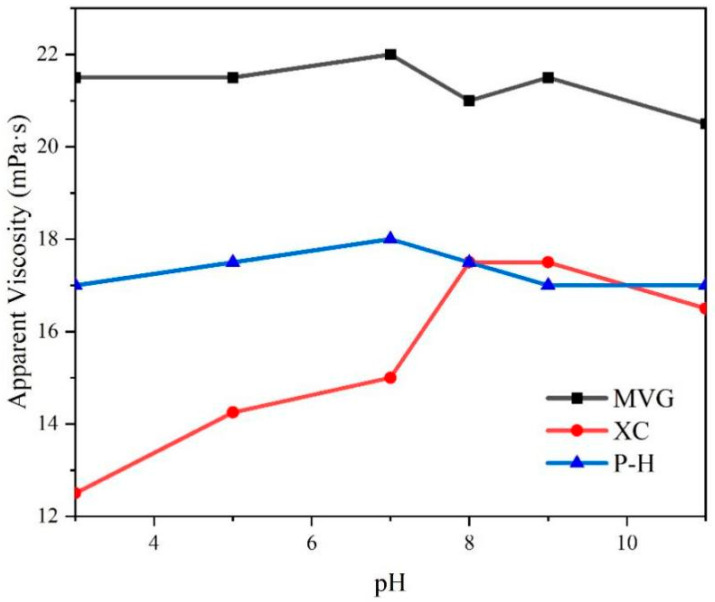
Apparent viscosity of the sea salt slurries containing natural high-molecular-weight polymers in a 0.5% concentration.

NaOH and HCl were added to sea water to adjust the pH, and then the three natural macromolecular polymers were added to obtain 0.5% aqueous solutions. We tested the viscosity of these solutions and found that the pH had a little effect on the viscosity of MVG and P-H solutions, with viscosity fluctuating by ±1.5 mPa·s. The viscosity of the solution containing XC varied significantly with the pH. In acidic sea salt water, the AV of the XC solution was significantly reduced. At pH < 8, AV increased with the increase in pH. At pH > 8, AV showed a downward trend. However, compared with a solution at neutral pH, the AV of the XC solution in alkaline conditions was larger than in neutral conditions, The specific results are shown in Figure 3. This indicated that alkaline conditions increase the viscosity of XC solutions.

### 2.6. Rheology and Stability of the Polymers in the Slurry at Different Dosages

**Figure 4:**
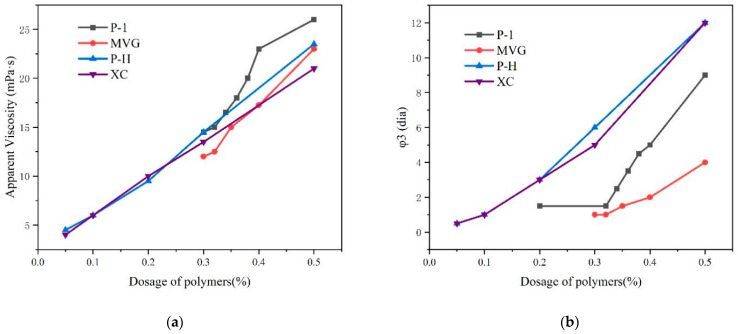
Apparent viscosity (**a**) and Φ3 (**b**) values of sea salt slurries containing the polymers at different concentrations.

We compared the viscosity and stability of P-1, XC, MVG, P-H, in a sea water slurry and determined the minimum required amount of polymer and viscosity that could stabilize slurry. The results are shown in Figure 4. The four polymers were mixed with calcium bentonite and anhydrous sodium carbonate and then mixed with a 4% sea salt slurry. After 16 h, we examined the appearance of liquid–solid phase separation in the slurry. The slurry containing the P-1 polymer started to delaminate in the presence of 0.35% P-1. The slurries containing XC and P-H started to stratify in the presence of 0.05% polymers. The MVG-containing slurry started to stratify with 0.32% MVG. We found that the slurries containing XC and P-H had the best stability, which could be maintained with in very small amounts of these polymers. MVG and P-1 appeared inferior to XC and P-H in this respect.

### 2.7. Description and Properties of the Composite Salt-Resistant Polymer Tackifier SNV 

**Figure 5:**
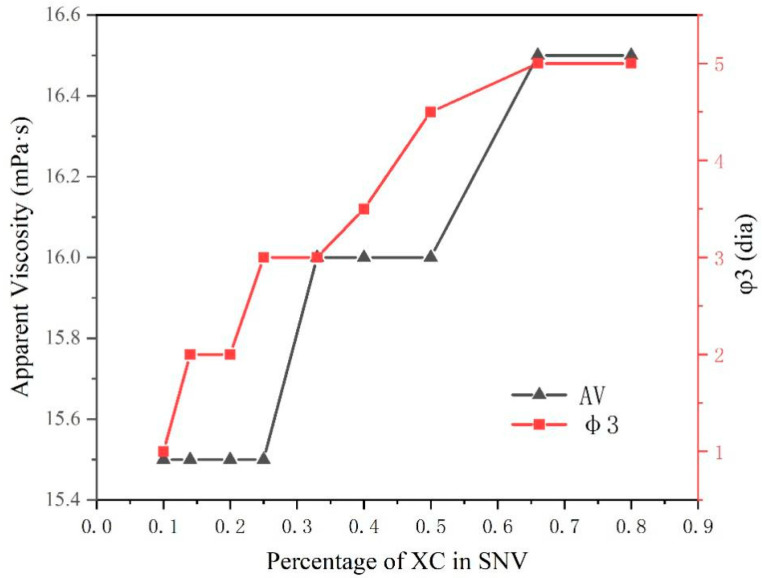
Apparent viscosity and Φ3 values of SNV bentonite slurries with different ratios of the two slurry components.

The viscosifying effect and ability to maintain slurry stability of the eight polymers were compared. We then combined MVG and XC in different proportions, obtaining a composite that we named SNV. We added 0.3% SNV and 4% anhydrous sodium carbonate to calcium-based bentonite and then mixed the resulting combination with sea salt water to test its rheology and stability. The specific results are shown in Figure 5.

The viscosity of SNV (MVG–XC), with different rations of the two components, did not change significantly in sea salt water. The value of φ3 increased when the XC ratio increased. In Table 3, the AV and YP of XC and MVG are similar; therefore, the viscosity changed little after combining the two polymers. The φ3 value of the XC slurry was larger; the φ3 value reflects the gel strength of a suspension. We observed that by increasing the XC ratio, the gel stability of the bentonite suspension improved.

### 2.8. Rheological Properties of the SNV–Bent Gel in Brine

In order to describe the rheological properties of the SNV–Bent gel in brine and the effect of the SNV composite polymer on the fluid parameters, the Power Law and Bingham Plastic and Herschel–Bulkley models were used [32].

Power Law:

(1) $$\tau ={K\gamma }^{n}$$

Bingham Plastic model:

(2) $$\tau =\tau_{0}+\mu_{p}\gamma$$

Herschel–Bulkley model:

(3) $$\tau =\tau_{y}+{K\gamma }^{n}$$

where Τ is the shear stress; γ is the shear rate; τ_0_ is the yield point; μ_p_ is the plastic viscosity; K is the consistency index; n is the fluidity index.

**Figure 6:**
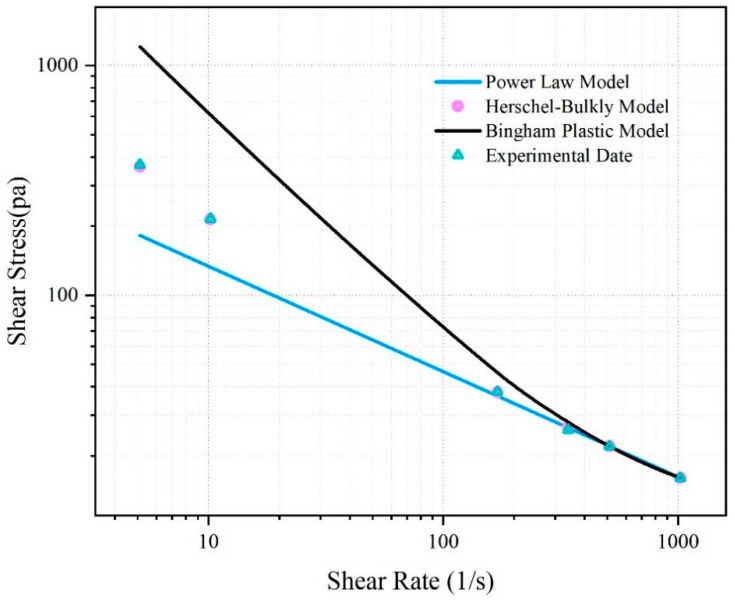
Rheological model of shear rate and viscosity of the SNV–Bent gel system.

**Figure 7:**
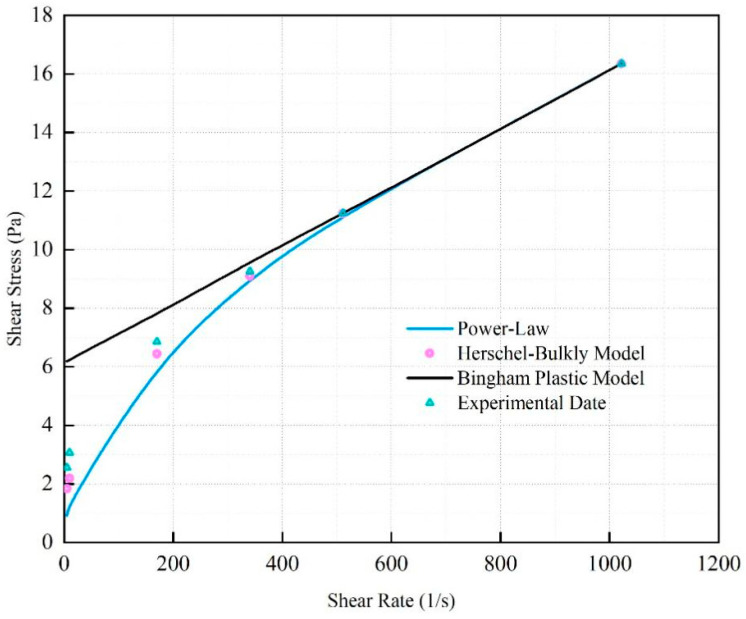
Rheological model of shear rate and shear strength of the SUV–Bent gel system.

The rheological curve of the fitted SNV–Bent gel in brine is shown in Figure 6. The fluid viscosity decreased with increasing shear rate. The SNV–Bent gel system is a kind of pseudoplastic fluid. The rheological curve of the SNV–Bent gel system shown in Figure 7 is the closest to that of the Herschel–Bulkley model, with R2 = 9.992. We found that the model could accurately calculate the shear stress. The fitting degree between the experimental data and the API standard curve was high, indicating that the drilling fluid of the SNV–Bent gel system conforms to the API standard.

**Figure 8:**
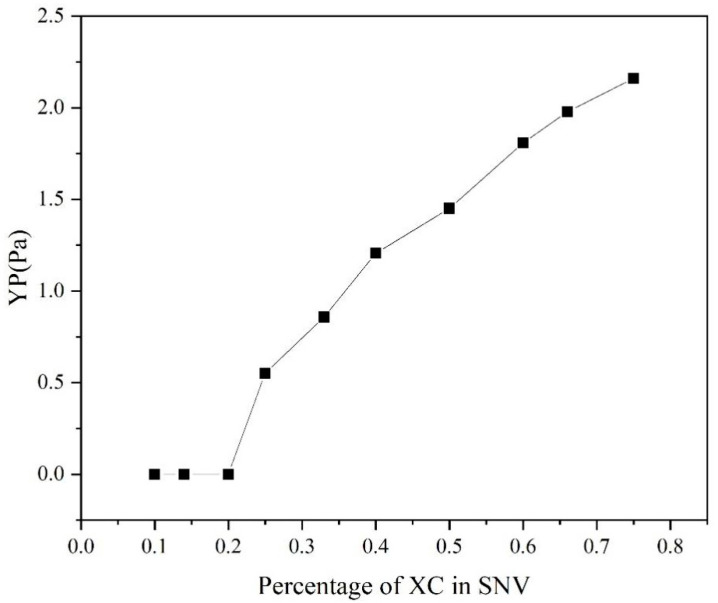
YP of the SNV–Bent systems with different ratios of the two components.

The $\tau_{0}$ of SNV–Bent gels with different MVG/XC ratios was calculated by the Herschel–Bulkley model, as shown in Figure 8. It can be seen that when the XC ratio was ≤1/5, $\tau_{0}$ was 0. When the XC ratio was 1/10, the SNV–Bent gel system was prone to solid–liquid phase separation. With the increase in XC addition, $\tau_{0}$ also increased, and the stability of the gel system also increased. Considering that $\tau_{0}$ reflects the stability of the gel system, the results showed that the larger the value of $\tau_{0}$, the greater the gel stability. The addition of XC could effectively improve the gel stability and, together with MVG, improved the slurry rheology [33].

### 2.9. SNV Usage and Economy

In brine, cations will enter the bentonite through ion exchange, which will reduce the viscosity and shear force of the drilling fluid; the clay particles will coalesce and settle. Therefore, SNV can be mixed with bentonite to prepare the salt-resistant bentonite STB and the salt-resistant bentonite slurry STM. The preparation was as follows: we adjusted the ratio of MVG and XC to obtain SNV and then mixed SNV with bentonite and sodium carbonate to directly obtain the salt-resistant bentonite STB. The prepared STB was then directly stirred and mixed with seawater to prepare STM.

**Figure 9:**
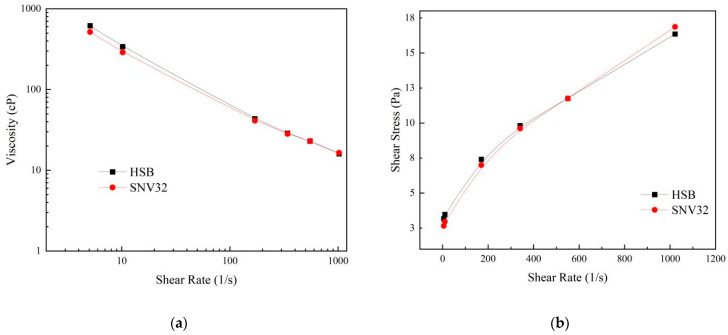
Comparison of SNV and HSB. (**a**) Shear rate and viscosity curves, (**b**) shear rate and shear strength curves.

We compared the commonly used product HSB with SNV32 (the SNV with the best performance, which we named SNV32). Its rheological curve is shown in Figure 9 [34]. The rheological curve of SNV32 at a 0.35% dosage was basically consistent with that of HSB at a 0.5% dosage. This showed that SNV32 can replace HSB, obtaining the same rheological properties but at lower dosage. The price of SNV32 at the concentration of 0.35% is much lower than that of HSB, and the use of SNV is very convenient. The combination of bentonite and SNV can be directly mixed with seawater. Compared with the traditional fresh water mixing method, the mixing steps and time are thus reduced. Therefore, SNV is a highly economical bentonite modifier.

## 3. Conclusions

Calcium-based bentonite cannot be fully hydrated and dispersed in salt water, which seriously affects its application in salt water drilling fluid. Sepiolite and palygorskite (attapulgite) clay minerals can be fully dispersed in brine, yielding high-viscosity brine suspensions. However, they cause a high loss in filtration, as they lead to the formation of a very thick filter cake; since the cost of reducing this filtration loss is very high, they are not suitable for the preparation of brine drilling fluids.

The biopolymer xanthan gum, XC, is the best salt resistant polymer among all commonly used water-soluble polymers, but its high price and low temperature resistance limit its application in high-temperature drilling fluids. The modified vegetable gum MVG, which is mainly made of an extensively available vegetable gum with a relatively low price, has high salt resistance after crosslinking. The polymer thickener SNV prepared by combining MVG with the biopolymer XC has a convenient cost/performance ratio and is suitable for use in brine and even seawater drilling fluids.

SNV improves the rheological properties of a calcium-based bentonite slurry and the dispersion stability of bentonite. Using SNV at a dosage of 0.35%, the apparent viscosity (AV) of the base slurry increased from 2 mPa·s to 32 mPa·s. The gel strength at 3 rpm increased from 0 dia to 5 dia. This greatly improved the viscosity and cuttings carrying capacity of the bentonite drilling fluid. The bentonite drilling fluid prepared with SNV can be directly slurried with brine and even seawater drilling fluids when drilling in ocean, coastal saline water and high-salinity surface saline water areas, reducing the slurry preparation cost and time.

## 4. Materials and Methods

### 4.1. Materials

The calcium-based bentonite used in the experiment was provided by Tianjin Dagang Oilfield and was of technical grade. The natural sea salt used was provided by Shandong Pengzhan Chemical Co., Ltd. (Shandong, China), and was of industrial grade.

**Figure 10:**
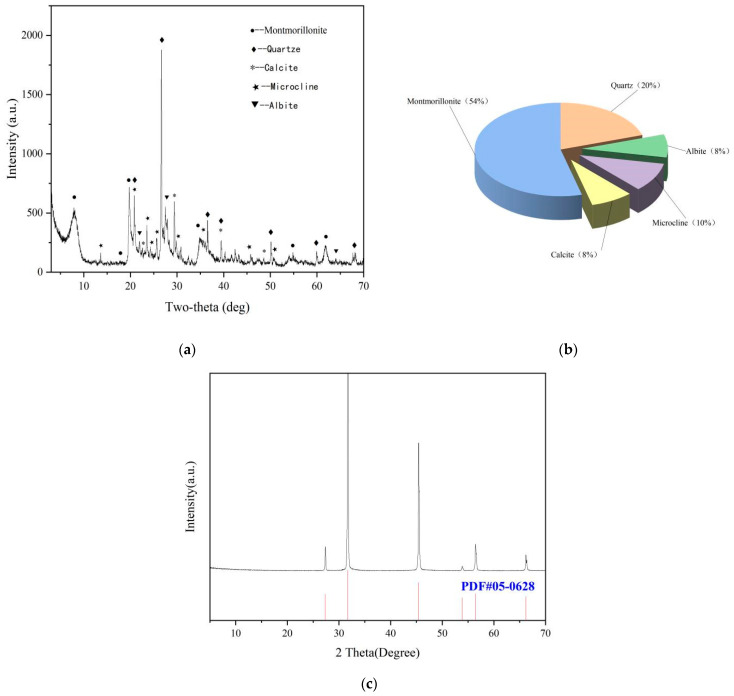
(**a**) XRD results of calcium bentonite (35 KV (30 mA)^−1^, 8 °min^−1^, 0.02 °step^−1^), (**b**) main chemical components of calcium bentonite, (**c**) XRD results of sea salt (35 KV (30 mA)^−1^, 8 °min^−1^, 0.02 °step^−1^).

The chemical and physical compositions of bentonite and natural sea salt were analyzed by X-ray diffraction (XRD) are shown in Figure 10. The suspension performance and rheological properties were measured with an ZNN-D6 six-speed rotational viscometer, a high-speed mixer, an electronic balance, a measuring cylinder. The above instruments were provided by Germany Bruker (Beijing) Technology Co., Ltd. (Beijing, China), model (D8 Advance).

Both P-1 (a kind of amphoteric polymer) and P-H (a kind of natural modified polymer) used in the experiments were provided by Shandong Juxin Chemical Co., Ltd. (Zibo, China), and were of industrial grade. PAS (polycarboxylate water reducer) was provided by Beijing Times Motion Technology Co., Ltd. (Beijing, China), and was of industrial grade. XC (xanthan gum) was provided by Ordos Zhongxuan Biochemical Co., Ltd. (Ordos, China), and was of industrial grade. CMC (carboxymethyl cellulose) and HMPC (hydroxypropyl methyl cellulose) were purchased from Sinopharm Chemical Reagent Beijing Co., Ltd. (Beijing, China), and were of CP grade. A7 was self-synthesized in the laboratory. HSB was supplied by domestic manufacturers and was of industrial grade. Chemical reagents such as distilled water and anhydrous sodium carbonate used in the experiments were purchased from Sinopharm Chemical Reagent Co., Ltd., and were of CP grade. SNV is a series of products obtained by mixing XC and MVG in different proportions.

### 4.2. Methods

#### 4.2.1. Preparation of the Synthetic Polymer A7

AM, AMPS, AA were dissolved in distilled water, and the pH of the solution was adjusted with NaOH to neutrality. The neutral solution was added to a three-necked flask, stirred, and heated to 55 °C, while passing nitrogen. A certain amount of potassium persulfate and sodium bisulfite was weighed and added to the heated solution to start the reaction. After 5 h, the reaction was completed, and a gel-like polymer was obtained. It was placed into an oven to be dried at 95 °C, pulverized and sieved to obtain the powder A7.

#### 4.2.2. Preparation Method of the Polymer Bentonite Suspension and Polymer Test Method

Preparation method of the bentonite suspension: Weigh 14 g of sea salt and dissolve it in 350 mL of distilled water to prepare 4% seawater. Weigh a certain amount of polymer (depending on the proportion, 0.5% if not specified), 22.5 g of calcium-based bentonite and 0.9 g of anhydrous sodium carbonate, mix thoroughly, pour into sea salt water and stir at high speed for 20 min; the stirring speed was 11,000 ± 300 r/min. After stirring, the container was sealed and maintained at room temperature for 16 h. To prepare the blank control base slurry, the polymer was not added.

Suspension performance test method: After 16 h of refinement, we observed and recorded the stratification of the suspension. Then, we poured the suspension into a high-speed stirring cup and stirred at a speed of 11,000 ± 300 r/min for 5 min. The readings at each rotational speed were recorded with a six-speed rotational viscometer.

Suspension rheological properties and stability evaluation methods: The performance of the bentonite suspensions as drilling fluids were evaluated by rheological data. Commonly used parameters are apparent viscosity (AV), which is commonly used to describe slurry fluidity, plastic viscosity (PV), which reflects the strength of internal friction between suspended solid particles, solid particles and liquid phase, and continuous liquid phase when the destruction and recovery of the mesh structure in a drilling fluid are in dynamic equilibrium under laminar flow, dynamic shear (YP), which reflects the strength of the internal gel network, yield point and ratio of plastic viscosity (RYP), which indicates the shear dilution of the drilling fluid as it flows. They were calculated using a six-speed rotational viscometer. Stability was determined by direct visual inspection of the layering of the suspensions.

(4) $$\mathrm{AV}=0.5\times R_{600}$$

(5) $$\mathrm{PV}=R_{600}-R_{300}$$

(6) $$\mathrm{YP}=0.511\times \left(R_{300}-\mathrm{PV}\right)$$

(7) RYP=YP/PV

AV—apparent viscosity, mPa·s;
PV—plastic viscosity, mPa·s;
YP—yield point, Pa;
RYP—ratio of yield point to plastic viscosity, Pa/mPa·s;
R_600_—viscometer dial reading at 600 r/min, dia;
R_300_—viscometer dial reading at 300 r/min, dia.

## Figures and Tables

**Table 1:** Rheology and stability of the slurry after the addition of cellulosic polymers.

Additives	AV (mPa·s)	PV (mPa·s)	YP (Pa)	RYP (Pa/(mPa·s))	Settling Behavior
Base slurry	2	1	1	1	Y
CMC	24.5	15	8.5	0.57	Y
HPMC	41	20	21	1.05	Y

**Table 2:** Rheology and stability of the slurry after adding the examined synthetic polymers.

Additives	AV (mPa·s)	PV (mPa·s)	YP(Pa)	RYP (Pa/(mPa·s))	Settling Behavior
Base Slurry	2	1	1	1	Y
P-1	26	17.5	8.5	0.49	N
PAS	2.5	2	0.5	0.25	Y
A7	16	13	3	0.23	N

**Table 3:** Rheology and stability of the slurries after adding the examined natural polymers.

Additives	AV (mPa·s)	PV (mPa·s)	YP (Pa)	RYP (Pa/(mPa·s))	Settling Behavior
Base Slurry	2	1	1	1	Y
XC	21	9	12	1.3	N
P-H	23.5	10	13.5	1.3	N
MVG	23	9	14	1.5	N

## Data Availability

Data are available from the authors upon request.
